# Proteomics dataset of epididymal fluid, seminal plasma, and proteins loosely attached to epididymal and ejaculated sperm from Angus bulls

**DOI:** 10.1016/j.dib.2022.108150

**Published:** 2022-04-15

**Authors:** Saulo Menegatti Zoca, Emmalee J. Northrop-Albrecht, Julie A. Walker, Robert A. Cushman, George A. Perry

**Affiliations:** a Department of Animal Science, South Dakota State University, Brookings, SD 57007, USA; b USDA, ARS, Roman L. Hruska US Meat Animal Research Center, Clay Center, NE 68933, USA

**Keywords:** Bull, Ejaculation, Epididymis, Protein, Spermatozoa, Sperm longevity

## Abstract

Peptides and proteins were identified by liquid chromatography with tandem mass spectrometry analysis (LCMS-MS) on an Orbitrap Velos mass spectrometer to further understand biological mechanisms that regulate increased longevity in epididymis compared to ejaculated sperm. Semen from sexually mature bulls were collected and then bulls were slaughtered to collect epididymal samples from the cauda epididymis. All samples were centrifuged to separate spermatozoa from fluids. A high ionic solution was used to remove surface proteins from spermatozoa. Four unique samples were generated: (1) epididymal fluid, (2) seminal plasma (ejaculated fluid), and proteins stripped from (3) epididymal sperm, and (4) ejaculated sperm. Samples were analyzed by LCMS-MS, and data were interpreted with Protein Pilot 5. False discovery rate (FDR) was set at 1%. Unique proteins (*n* = 458) were identified in ejaculated samples, 178 proteins in seminal plasma and 298 proteins stripped from ejaculated sperm. In epididymal samples, 311 proteins were identified in the fluid, and 334 were identified among proteins stripped from epididymal sperm. This dataset can be useful for further understand of biological mechanisms that control sperm longevity. This dataset is related to the article ‘Proteomic analyses identify differences between bovine epididymal and ejaculated spermatozoa that contribute to longevity’ by (Zoca et al., 2022).

## Specifications Table

**Table:** 

Subject	Biological Sciences: Animal Physiology
Specific subject area	Sperm biology; sperm maturation; association of epididymal and ejaculated fluids and loosely attached to the sperm proteins to sperm function
Type of data	Tables
How data were acquired	Liquid chromatography with tandem mass spectrometry analysis (LCMS-MS) on an Orbitrap Velos mass spectrometer.
Data format	Raw Filtered Analyzed
Description of data collection	Semen was collected from nine bulls. After a resting period to renormalize epididymal reserves bulls were slaughtered. Epididymides were dissected and epididymal fluid and spermatozoa were collected from the cauda epididymis. All samples were centrifuged to separate spermatozoa and fluids (epididymal fluid or seminal plasma). Spermatozoa were washed with a high ionic solution to remove proteins attached to the sperm surface. Samples were then centrifuged to separate stripped proteins from the spermatozoa.
Data source location	Open Prairie South Dakota State University Brookings, South Dakota United States
Data accessibility	Open Prairie repository. Article with all data and analysis etd2/221: https://openprairie.sdstate.edu/cgi/viewcontent.cgi?filename=2&article=1205&context=etd2&type=additional Supplemental file 1 identified proteins fluid – 3.1205 https://openprairie.sdstate.edu/cgi/viewcontent.cgi?filename=3&article=1205&context=etd2&type=additional Supplemental file 2 identified proteins sperm – 4.1205 https://openprairie.sdstate.edu/cgi/viewcontent.cgi?filename=4&article=1205&context=etd2&type=additional Supplemental file 3 Gene ontology fluid – 5.1205 https://openprairie.sdstate.edu/cgi/viewcontent.cgi?filename=5&article=1205&context=etd2&type=additional Supplemental file 4 Gene ontology sperm – 6.1205 https://openprairie.sdstate.edu/cgi/viewcontent.cgi?filename=6&article=1205&context=etd2&type=additional Supplemental file 5 KEGG analysis fluid – 7.1205 https://openprairie.sdstate.edu/cgi/viewcontent.cgi?filename=7&article=1205&context=etd2&type=additional Supplemental file 6 KEGG analysis sperm – 8.1205 https://openprairie.sdstate.edu/cgi/viewcontent.cgi?filename=8&article=1205&context=etd2&type=additional Mass spec raw data files 2.1205: https://openprairie.sdstate.edu/cgi/viewcontent.cgi?filename=2&article=1205&context=etd2&type=additional
Related research article	S. Menegatti Zoca, E.J. Northrop-Albrecht, J.A. Walker, R.A. Cushman, and G.A. Perry, Proteomic analyses identify differences between bovine epididymal and ejaculated spermatozoa that contribute to longevity, Theriogenology. 184 (2022):51-60. https://doi.org/10.1016/j.theriogenology.2022.02.021

## Value of the Data

• This dataset provides a comprehensive proteomic analysis of seminal plasma and epididymal fluid of bulls, as well as proteins attached to the sperm in the epididymis or during ejaculation. This dataset provides critical proteomic information related to bovine sperm function and its interaction with the environment.
• Sperm biologists and animal scientists can use this dataset as a basis for understanding the role of sperm proteins in bovine reproductive efficiency.
• This dataset can be useful for further understanding of biological mechanisms that control sperm longevity in the epididymis compared to ejaculated sperm, particularly in bovine.

## 1 Data Description

Liquid chromatography with tandem mass spectrometry analysis identified proteins in the fluid fractions (seminal plasma and epididymal fluid) and attached to sperm (ejaculated and epididymal). Supplemental files 1 and 2 show identified proteins and includes Protein name, species, official gene symbol, accession number, protein molecular weight, protein grouping ambiguity, spectra count, and differences between ejaculated and epididymal samples. In Supplemental file 3, there are 38 GO terms related to proteins identified in ejaculated fluid of which 26 had FDR < 0.05; there are 58 GO terms related to proteins present in epididymal fluid of which 26 had FDR < 0.05. There are also 30 GO terms that are present in both ejaculated and epididymal fluid. Supplemental file 4, contains 50 GO terms related to proteins stripped from ejaculated sperm of which 23 had FDR < 0.05; there are 56 GO terms related to proteins stripped from epididymal sperm. There are also 38 GO terms in common between samples from epididymal sperm and ejaculated sperm. Fluids and loosely attached sperm protein KEGG, count, protein gene symbol per KEGG and FDR are presented in Supplemental files 5 and 6, respectively.

## 2 Experimental Design, Materials and Methods

### 2.1 Experimental design

Semen (collected by electroejaculation) from 4-yr old sexually mature bulls (*n* = 9) that had previously successfully bred cows was collected weekly (2 ejaculates) and discarded. After one week of rest bulls were collected for the samples utilized in the analysis. Before being slaughtered, bulls were rested for six weeks (following third semen collection) to allow the epididymal reserve to renormalize. Testes and epididymides were collected at the local abattoir and transported back to the laboratory where the epididymis was dissected and epididymal fluid and spermatozoa were collected from the cauda epididymis [1].

### 2.2 Protein isolation

Spermatozoa were separated from fluids (epididymal fluid or seminal plasma) by centrifugation (700 × g for 10 min). The supernatant was removed, snap frozen in liquid nitrogen, and stored at -80 °C until analysis occurred. A high ionic solution was used to wash the spermatozoa [2] by vortexing for 1 min to remove loosely attached surface proteins. All samples were again centrifuged (700 × g for 10 min) and the supernatant was collected, snap frozen in liquid nitrogen, and stored at -80 °C until analysis occurred. In total four sample types were collected: (1) epididymal fluid, (2) ejaculated fluid, and proteins stripped from (3) epididymal sperm, and (4) ejaculated sperm.

### 2.3 Liquid chromatography mass spectrometry analysis

Samples from each of the nine bulls were pooled together by sample type [(1) epididymal fluid, (2) ejaculated fluid, (3) epididymal sperm stripped proteins (epididymal sperm), and (4) ejaculated sperm stripped proteins (ejaculated sperm)]. This resulted in one pool for each sample type. Samples were shipped to the University of Minnesota Mass Spectrometry facility, and liquid chromatography with tandem mass spectrometry was conducted there. Briefly, a 5 ug aliquot of each sample was mixed with 4X Laemmli buffer (final concentration 1X), heated at 95 °C for 5 min, and then loaded on an 8–16% BioRad Criterion™ Gel. The gel was run at 25 mA constant current for 30 min. The gel was stained with Imperial™ Protein Stain (ThermoFisher Scientific). The protein region for each sample was cut out, in-gel trypsin digested, and Stage Tip cleaned up as previously published [3] with the following change: the samples were alkylated with iodoacetamide. Peptides (∼400 ng of reconstituted peptides) were then analyzed on an Orbitrap Velos mass spectrometer [4] with the following modifications: (1) capillary column diameter was 100 µm, (2) gradient elution profile was of 8–35% B Solvent over 67 min at 330 nL/min [A Solvent was 98:2:0.01, H_2_O:acetonitrile(ACN):formic acid (FA); and B Solvent was 98:2:0.01, ACN:H_2_O:FA], (3) lock mass was not employed.

Dynamic exclusion settings were:

1. repeat count = 1,
2. exclusion list size was 200,
3. exclusion duration = 12 s,
4. exclusion mass width (high and low) was 15 ppm,
5. early expiration was disabled.

### 2.4 Database searching and criteria for protein identification

Sequest (Thermo Fisher Scientific, San Jose, CA, USA; version 2.1.0.81) was used to analyze all LCMS/MS data. The Uniprot.org bovine (taxid 9913) database of protein sequences was searched (downloaded on March 6, 2013). Both canonical and isoforms were included. Searched database was merged with a database containing the common lab contaminant proteins (thegpm.org/crap/index, 109 proteins). The search included: (1) trypsin as the digestion enzyme, (2) fragment ion mass tolerance was set at 0.100 Da, and (3) precursor tolerance was set at 50 ppm. Variable peptide modification included: (1) Oxidation and di-oxidation of methionine, (2) deamidated of asparagine, glutamine and pyroglutamic acid, and (3) N-terminal protein acetylation set as a variable modification and carbamidomethyl cysteine set as a fixed modification.

All LCMS/MS based peptide and protein identifications were validated with Scaffold (version Scaffold_5.0.0, Proteome Software Inc., Portland, OR). Peptide identifications were accepted at greater than 99.0% probability by the Scaffold Local FDR algorithm. Identified proteins that established greater than 7.0% probability, with an FDR less than 1.0%, and peptides identified were greater than or equal to 2 were accepted. Protein Prophet algorithm [5] was used to assign protein probabilities. The principles of parsimony were used to group proteins that contained similar peptides and could not be differentiated based on MS/MS analysis alone. Clusters were formed by grouping proteins sharing significant peptide evidence.

Total spectrum count was used to identify proteins that were present in each sample (≥ 0.8 peptides identified as exclusive and unique to each protein). The official gene name of proteins identified in each of the four sample types were entered into DAVID v 6.8 [6,7] with *Bos taurus* as background to determine the top Gene Ontology and Kyoto Encyclopedia of Genes and Genomes (KEGG) pathways (thresholds: count = 2, EASE = 0.1). The FDR for each Gene Ontology and KEGG was generated by DAVID v 6.8.

## Ethics Statement

All procedures were approved by the South Dakota State University Institutional Animal Care and Use Committee (#14-031A) and conformed to the Guide for the Care and Use of Agriculture Animals in Research and Teaching.

## CRediT authorship contribution statement

**Saulo Menegatti Zoca:** Formal analysis, Investigation, Writing – original draft, Writing – review & editing. **Emmalee J. Northrop-Albrecht:** Investigation, Writing – review & editing. **Julie A. Walker:** Writing – review & editing, Supervision. **Robert A. Cushman:** Conceptualization, Investigation, Writing – review & editing, Supervision. **George A. Perry:** Conceptualization, Methodology, Investigation, Resources, Writing – review & editing, Supervision, Funding acquisition.

## Declaration of Competing Interest

The authors declare that they have no known competing financial interests or personal relationships which have or could be perceived to have influenced the work reported in this article.

## Data Availability

Mass spec raw data files (Original data) (Open Prairie).

## Appendix Supplementary materials

Image, application 1

Image, application 2

Image, application 3

Image, application 4

Image, application 5

Image, application 6
